# Advanced Technology Use by Care Professionals

**DOI:** 10.3390/ijerph17030742

**Published:** 2020-01-23

**Authors:** Tom Brandsma, Jol Stoffers, Ilse Schrijver

**Affiliations:** 1 Research Centre for Employability, Zuyd University of Applied Sciences, 6131 MT Sittard, The Netherlands; tom.brandsma@zuyd.nl (T.B.); ilse.schrijver@zuyd.nl (I.S.); 2 Faculty of Management, Open University of the Netherlands, 6419 AT Heerlen, The Netherlands; 3 Research Centre for Education and the Labour Market (ROA), Maastricht University, 6211 LM Maastricht, The Netherlands

**Keywords:** technology acceptance, advanced technology, elderly care, performance expectancy, logfiles, longitudinal design

## Abstract

Advanced technology is a primary solution for the shortage of care professionals and increasing demand for care, and thus acceptance of such technology is paramount. This study investigates factors that increase use of advanced technology during elderly care, focusing on current use of advanced technology, factors that influence its use, and care professionals’ experiences with the use. This study uses a mixed-method design. Logfiles were used (longitudinal design) to determine current use of advanced technology, questionnaires assessed which factors increase such use, and in-depth interviews were administered to retrieve care professionals’ experiences. Findings suggest that 73% of care professionals use advanced technology, such as camera monitoring, and consult clients’ records electronically. Six of nine hypotheses tested in this study were supported, with correlations strongest between performance expectancy and attitudes toward use, attitudes toward use and satisfaction, and effort expectancy and performance expectancy. Suggested improvements for advanced technology include expanding client information, adding report functionality, solving log-in problems, and increasing speed. Moreover, the quickest way to increase acceptance is by improving performance expectancy. Care professionals scored performance expectancy of advanced technology lowest, though it had the strongest effect on attitudes toward the technology.

## 1. Introduction

Organizations in the health care industry are facing different challenges, like labor shortages and increasing demand for care and the use of technology could help care professionals to solve these challenges [1,2]. Thompson and Brailer [3] (p. 38) define technology in the field of health care as *“the application of information processing involving both computer hardware and software that deals with the storage, retrieval, sharing, and use of health care information, data, and knowledge for communication and decision making”*. This definition also incorporates recent advanced technologies like the mobile tablet computer in which care professionals can retrieve relevant information about their clients (i.e., the Electronic Health Record (EHR). According to Koutsouris and Lazakidou [4], the use of technology empowers care professionals to cope with the rising demand for care. Moreover, use of technology could increase the quality of care and decrease health care costs [5,6,7,8,9]. Although advanced technology is promising, it is not widely used and in care organizations because of negative attitudes towards technology, ambiguous expectations and needs, and organizational complications during the implementation [10,11]. Most research into acceptance and use of technology focus on hospitals (i.e., cure industry) [12,13]. However, research results in cure-industry results cannot be generalized to the care industry [13]. One significant difference between these industries is the education of the care professionals (i.e., end users) in them. The workforce regarding elderly care (i.e., care industry), for example, completes secondary vocational education and training courses at levels 1, 2, or 3 [14], and in hospitals (i.e., cure industry), most care professionals are trained at level 4 or graduate from higher professional education (i.e., level 5) [14]. According to Porter and Donthu [15], educational level is reported as an important individual factor in adopting technologies, which emphasize the difference between cure- and care professionals in this regard. Research especially in the care-industry could lead to a better understanding of the acceptation and use of technology by professionals in the NAH (i.e., nursing, auxiliary health care, and home care) industry. Therefore, this study investigates the acceptance and use of advanced technology by professionals working in the NAH industry. Recent developments in the field of advanced technology have led to the implementation of the mobile tablet computer within two care-providing organizations in the Netherlands. With this advanced technology, care professionals can access the Electronic Health Record (EHR), using cameras to monitor clients and get access to relevant care-protocols. For care professionals these functionalities play an important role enhancing their care delivery process more effective and efficient. Although the implementation of these technologies should be determined by serviceability for end users, so far little consideration is given to care professionals’ reactions and experiences [12,16]. Little is known about care professionals’ perceptions regarding advanced technology, its usability, and factors that influence use [17,18]. Therefore, the central research question of this study is how the acceptance and use of advanced technology by care professionals in the elderly care can be enhanced. To address this research question, a three-component study was conducted: (a) the actual use of the advanced technology, (b) explanatory factors to technology acceptance and (c) reactions and experiences of the use of advanced technology. This study tracks use of advanced technology using log files, and since such files contain a year’s worth of data, this study uses a longitudinal design, which is uncommon during technology acceptance research [19]. The actual use of such technology is not commonly measured, and studies often use subjective methods during data collection, such as self-reports [12,20,21]. Objective user data might lead to insights into both use and the relationship between affective and behavioral responses [10,22]. Besides, most studies on technology acceptance focus on a quantitative design [23], and in order to get a better understanding of actual and employees’ opinions of technology use, this study also uses semi-structured interviews. This study is relevant practically, since findings are translatable into applications that improve technology use.

## 2. Theoretical Foundations

### 2.1. Acceptance Theories and Models

In recent years, many theories, models, and assessments have appeared in the literature to explain acceptance and use of advanced technology. One is the technology acceptance model (TAM), developed by Davis [24], which is based on Fishbein and Ajzen’s [25] theory of reasoned action (TRA). In the TAM, perceived usefulness and ease of use are theorized to influence affective responses, behavioral intentions, and observable behaviors (e.g., technology use). Venkatesh et al. [26] describe and compare eight technology acceptance theories and models, including the TRA and TAM. Drawing from these models, the authors introduced the unified theory of acceptance and use of technology (UTAUT), which suggests that performance expectancy, effort expectancy, social influence, and facilitating conditions predict behavioral intentions and thus observable behaviors. Performance expectancy represents the degree to which a user believes that technology helps achieve better work results. Effort expectancy is the degree to which a user finds a technology easy to use. Social influence represents the degree to which a user experiences social pressures to use technology. Facilitating conditions are the extent of a user’s perceptions regarding whether the organization facilitates circumstances to use technology [23,26]. The variables performance expectancy and effort expectancy of the UTAUT are equal to the variables perceived usefulness and perceived ease of use of the TAM [27,28]. In addition to the TAM, the contextual factors (facilitating conditions and social influence) are also included in the UTAUT [29]. In addition, according to Venkatesh [26], the UTAUT appears to be an adequate framework to explain technology acceptance, more specific in the context of the care industry [23,27,30]. Therefore, in this study, the UTAUT will be used as the basic research model to investigate technology acceptance by care professionals.

### 2.2. Towards the Construction of a Research Model, Based on the UTAUT

In line with the basic UTAUT, we argue that a positive relationship exists between facilitating conditions and actual use of technology [26,29], since care professionals working in elderly care require adequate assistance and support in the use of technology [31]. Thus:

**Hypothesis** **1** **(H1)** Facilitating conditions and use behaviors correlate positively.

According to Kao [28] performance expectancy is important with regard to the adoption of technology and has a significant relationship with affective behavior [27]. More specifically, multiple studies report an influence of performance expectancy on attitudes [32,33]. Performance expectancy also has a positive influence on care professionals’ attitudes in the context of health care [8,10]. When care professionals feel that technology contributes to the improvement of the quality of care given and leads to efficient work processes, they are inclined to use technology far more than when it disrupts workflows. Nictiz [31] found that care professionals working in health care attach value to the reliability and proper operation of technology, both from technical (i.e., the technology must work properly) and functional (i.e., how technology helps during work) perspectives [31]. If care professionals perceive technology as reliable and functional, they develop positive attitudes towards it. Therefore:

**Hypothesis** **2** **(H2)** Performance expectancy and attitudes correlate positively.

Kim et al. [10], Aggelidis et al. [8] and Hsieh [27] show that effort expectancy influences care professionals’ attitudes, findings that are supported in a recent meta-analysis by Dwivedi et al. [29]. Care professionals who found technology difficult to use, and consequently had negative attitudes toward it, developed workarounds to avoid using it. Thus:

**Hypothesis** **3** **(H3)** Effort expectancy and attitudes correlate positively.

Aggelidis and Chatzoglou [8] and Maillet et al. [1] found a direct positive link between effort expectancy and performance expectancy. When care professionals found technology easy to use, they believed it added value to their work, and thus:

**Hypothesis** **4** **(H4)** Effort expectancy and performance correlate positively.

According to Bandura [34], social influence plays a role in human behavior and decision making. Moreover, it influences affective behavior [27]. A meta-analysis by Dwivedi et al. [29] found that individuals adjust their attitudes based on other people’s information and stories [29,33]. Therefore:

**Hypothesis** **5** **(H5)** *Social influence and attitudes correlate positively*.

In addition, to the original UTAUT, in this study, computer self-efficacy was added to the research model as an independent variable, defined as ‘an individual perception of his/her ability to use technology in the accomplishment of a task’ [8] (p. 117). The individual context to the UTAUT is expected to explain an even greater portion of the variance during technology acceptance [8,35]. Numerous studies report that computer self-efficacy influences effort expectancy [1,8]. According to O’Leary [36], perceptions motivate people to engage in behaviors. When care professionals have little confidence in their ability to use technology, their attitudes toward its use diminishes in comparison to when they have greater computer self-efficacy. Studies in the health care industry suggest a positive relationship between computer self-efficacy and attitude [37]. Thus:

**Hypothesis** **6** **(H6)** *Computer self-efficacy and attitudes correlate positively*.

Maillet et al. [1] found a direct positive relationship between computer self-efficacy and effort expectancy; the greater a care professional’s computer self-efficacy (i.e., self-confidence), the more positive the effort must be to use technology. Therefore:

**Hypothesis** **7** **(H7)** Computer self-efficacy and effort expectancy correlate positively.

Behavioral intentions were not included, since this study does not assess users’ intentions, but observable use of technology [1]. According to Fishbein and Ajzen [25], behavioral intentions are about future behaviors (i.e., pre-implementation stage), but in this study, behaviors (i.e., use) were already present (i.e., post-implementation stage). The UTAUT [26] comprises cognitive, affective, and behavioral components. Removing behavioral intentions from the original UTAUT means that the affective component is no longer represented in the conceptual model. To maintain the model’s integrity, in this research model, we include attitudes as an affective response, following Fishbein and Ajzen [25]. In a context in which staff members are required to use advanced technology, as is the case in the two organizations, Brown et al. [38] recommend that it is better to measure attitudes than intentions. This was the reason behind the second adaptation—addition of attitudes toward use. In line with the original UTAUT and other acceptance models such as the TAM and TRA, this study suggests a direct positive relationship between the affective component (i.e., attitudes) of these models and actual use [26]. Dwivedi et al. [29] and Hsieh [27] found a positive relationship between affective behavior and actual use, and thus:

**Hypothesis** **8** **(H8)** Attitudes and use behaviors correlate positively.

Satisfaction was added as a dependent variable because if technology is already in use, a user’s satisfaction should be assessed [1], which accords with Brown et al.’s [38] recommendation of measuring satisfaction when technology use is obligatory. Maillet et al. [1] found a positive relationship between care professionals’ use behaviors and satisfaction; greater use leads to greater satisfaction with use of a device. Thus:

**Hypothesis** **9** **(H9)** Use behaviors and satisfaction correlate positively.

**Figure 1:**
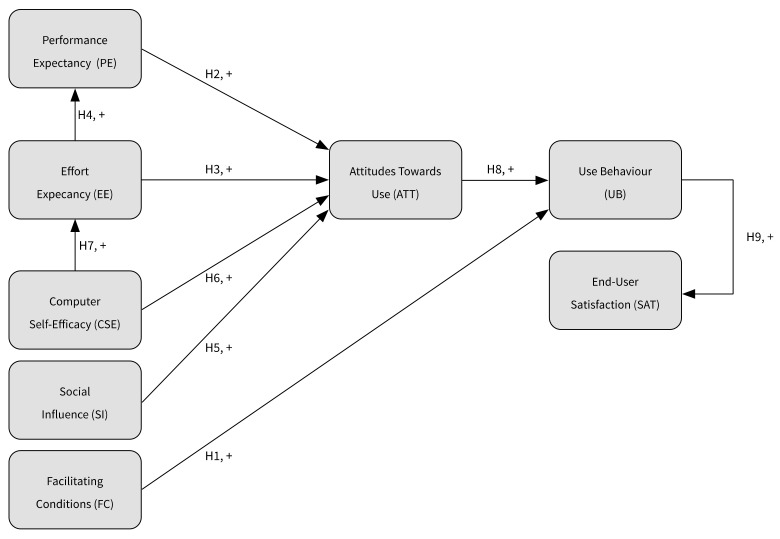
The constructed research model based on the unified theory of acceptance and use of technology (UTAUT). + positive relationship.

After the above-mentioned adjustments, the constructed research model of this study is presented below in Figure 1.

## 3. Methods

### 3.1. Design and Context

Two Dutch care organizations, providing residential and home care for the vulnerable elderly were selected for this study. The advanced technology was small, portable devices installed in residents’ rooms (i.e., bedside terminals) such that portability was eliminated. Organization A comprises 9 residential locations, with a workforce of 462 fulltime employees. Between 2013 and 2018, the organization implemented devices in stages across all locations, and all 9 locations were assessed during this study. Organization B comprises 17 residential locations, with a workforce of 1692 fulltime employees. Between 2011 and 2016, the organization implemented devices in stages across 8 locations, all of which were included in the study. This study uses a mixed-methods design, including both quantitative (i.e., questionnaires and log files) and qualitative (multiple case study with semi-structured interviews) data [39].

To prevent biases associated with lack of blinding for component 1 of this study we extracted anonymous logfile data from the advanced technology system before starting component 2 (questionnaires) and 3 (in-depth interviews) of this study. For a summarized overview of the research methods used, see Table 1.

### 3.2. Methods for Component Study 1: Observable Use

The first component study comprised quantitative, descriptive secondary data analysis, providing insights into use of the devices in terms of frequency (i.e., use volume) and diversity (i.e., functionalities). Log data were retrieved from a database from suppliers of the devices. The files, which were used to establish user patterns over a longer period, contained user information per care professional at all residential locations of both organizations. We selected 4 measurement periods (T1 to T4), from June 2017 to March 2018, each with a duration of one week (Monday to Sunday). To obtain reliable representations of regular device use, the periods did not coincide with vacations and holiday seasons. Sixty-four log files were anonymized by both organizations by replacing staff members’ names with staff numbers. For each location, the files were categorized into 4 timeframes for each period, with categorization based on usual time schedules in both organizations—morning (07:00 to 11:00), afternoon (11:00 to 16:00), evening (16:00 to 23:00), night (23:00 to 07:00). Anonymized HR files that contained the work hours, contract hours, sex, age, and positions of each staff number were linked with the log files. To determine the number of unique users of the devices, only users who were identifiable by staff numbers were analyzed.

### 3.3. Methods for Component Study 2: Testing the Research Model

To test the hypotheses, a second component study used a quantitative, cross-sectional design [39], during which the population (*n* = 1041) of care professionals in both organizations was administered questionnaires. Participation in this study was voluntary and semi-anonymous, since the care professionals were asked to report their staff numbers so they could be paired with the log files later (Component study 1). To maximize the response rate, location managers encouraged the care professionals to complete the questionnaire. An introductory letter was also included with the questionnaire, emphasizing that responses would never be traceable to individual staff members. For further enticement, 4 gift vouchers were raffled among respondents.

To measure performance expectancy, effort expectancy, social influence, facilitating conditions, computer self-efficacy, and attitudes toward use, the UTAUT questionnaire developed by Venkatesh et al. [26] was used. Nineteen questions assessed using a 7-point, Likert-type scale were collected at the individual level. The original UTAUT questionnaire was modified according to context and the devices defined as advanced technology under investigation. A questionnaire from Maillet et al. [1] was used to measure satisfaction, which included one question assessed using a 7-point, Likert-type scale. The original UTAUT questions were written in English and, using a forward–backward method [40], they were translated into Dutch. Beta testing of the questionnaire was performed with 3 care professionals from the sample. The final questionnaire was administered to the entire care personnel sample. Data were analyzed by using IBM’s statistical package SPSS. Behaviors (i.e., use) were measured using log files. Each respondent’s login frequency was determined using a staff number. Average use per working day was calculated by dividing login frequency by the number of scheduled days. Use was measured as a continuous variable and based on data from the first component study. Measuring use and attitudes independently increased reliability of the correlation [41].

Reliability of the scales was assessed using Cronbach’s alpha coefficient. All variables were ordinal but were included at the interval level so that they could be used during multiple analyses. Correlations between variables were assessed using Spearman’s rank-order correlation coefficient (rs), since the variables were not normally distributed [42]. For independent variables, we tested for multicollinearity, and following Venkatesh et al. [26], regression was conducted using scale averages. Although the variables correlated, the correlations did not exceed the threshold of 0.90, so there was no evidence of multicollinearity and the independent variables could be included separately during regression analysis [43].

### 3.4. Methods for Component Study 3: Care Professionals’ Experiences

Semi-structured interviews were used to track care professionals’ user experiences, since this study is exploratory in terms of outlining experiences and needs [39]. The interviews were administered at participants’ workplaces. Based on the questionnaires from Component Study 2, 79 of 180 care professionals indicated that they were willing to participate in an interview. We selected a random sample of six care professionals from this pool, [39] based on their satisfaction with the devices—unsatisfied (scores 1–3), satisfied (5–7), and neutral (4). The interviews covered several topics, including (a) general experience with the devices, (b) positive aspects of the devices, (c) issues for improvement of the devices, (d) facilitating circumstances, (e) influence on the work process, (f) location-wide functionalities, (g) number of logins, and (h) protocols. Near the conclusion of the interview, respondents were asked whether they had any other topics to discuss. Facilitating circumstances was included because during Component Study 2, the construct was found unreliable. Venkatesh et al. [26] and Dwivedi et al. [29] argue that this aspect is important during use of technology, so we examined it qualitatively. Location-wide functionalities (f), number of logins (g), and protocols (h) were selected based on results from Component Study 1:

- location-wide functionalities were nearly never used (e.g., cameras);
- the number of logins was higher than the number of functionalities used;
- protocols were rarely used, and following a pilot study, the organizations had questions about the added value of this functionality.

Interviews lasted between 16 and 46 min, with 30 min the average. After the fifth interview, data saturation was achieved [39], and transcripts were coded based on the topic list using open and selective coding [39]. Qualitative coding procedures follow grounded theory [44], based on an inductive perspective. However, according to Timmermans and Tavory [45] the most qualitative studies are not completely inductive but can be considered as abductive. Also, in this study, we add knowledge to the existing literature, combined with new insights (inductive). Therefore, this study can be labelled as abductive.

## 4. Results

### 4.1. Results of Component Study 1: Actual Use

#### 4.1.1. Information About Users of the Devices

Based on the schedules at both organizations, 1286 care workers were scheduled over the entire measurement period. The number of unique device users over the total measurement period was 933; 73% of care workers used the devices, which means 353 were non-users (27%). No differences regarding use were found between the organizations. Regarding missing data, 55 values for age and contract hours were missing at care organization B, which explains the difference between 434 and 489. Moreover, 18 values for gender were missing at care organization B, which explains the difference between 471 (428+43) and 489. As a result (in Total) these missing data explain the difference between 915 (845+70) and 933. User characteristics appear in Table 2.

#### 4.1.2. Information About Actual Use of the Devices

The devices were used an average of 5695 times (100%) over the measurement period and sample. They were used an average of 2548 (45%) times in the morning and 389 (7%) times in the afternoon. In the evening, the devices were used 1079 (19%) times, and 1679 (29%) times at night. Daily use by unique device users (*n* = 933) averaged six times each day. A decrease in the number of logins among the entire sample was observed at T1, followed by an increase at T4. In both organizations, the devices were most frequently used in the morning; night and afternoon came in second and third. Regarding functionalities that the devices controlled, a theme emerged. On average and for each day over the entire measurement period, local client cameras were most frequently used (3185 times), especially at night (2583). Electronic client records were consulted an average of 790 times per day, especially in the morning (561 times). The call-and-response logging functionality was used an average of 219 times per day, especially at night (143 times). Peripheral cameras were also consulted an average of 204 times a day, especially at night (169 times). Entry cameras were consulted 49 times per day, and more so at night (23). Location-wide functionalities were unused. Based on figures for the entire sample, each staff member used functionalities 4.75 times each day, and no functionalities were used during some log events. Few differences were observed between the organizations (Table 3).

### 4.2. Results of Component Study 2: Testing the Research Model

#### 4.2.1. Sample Demographics

One hundred and eighty of 1041 care professionals completed the questionnaire—85 of whom worked for Organization A and 85 for Organization B—with a response rate of 17.3%. The entire response group comprised 14 men (8%) and 166 women (92%). The oldest respondent was 66 and the youngest 17. Respondents’ average age was 46, and the average number of working hours was 26, with a minimum of 2 and maximum of 36. Most respondents worked as individual health care assistants, level 3 (76%). The proportions of sex, average age, part-time positions, and division of jobs (level 3) were typical of that in elderly care [14]. Respondents had an average of 26 months experience with the devices, with a minimum of zero and maximum of 180.

#### 4.2.2. Reliability of the Constructs and Their Correlation

Internal consistency of the scales was assessed using Cronbach’s alpha coefficient. Performance expectancy (0.859), effort expectancy (0.879), social influence (0.817), and attitudes toward use (0.906) exceeded 0.7 and were therefore reliable [42]. Although there is consensus on the threshold of 0.7 [42], some researchers argue that a lower threshold is acceptable [46,47]. A threshold of 0.6 is justified, especially when questions are used in a new context [48]. The reliability for facilitating conditions was inadequate (0.555), even after deletion of items, and was thus excluded from analysis. Computer self-efficacy had a coefficient of 0.594 and was included. Table 4 shows an overview of the Cronbach’s alpha coefficients.

#### 4.2.3. Correlation and Regression Analysis

**Figure 2:**
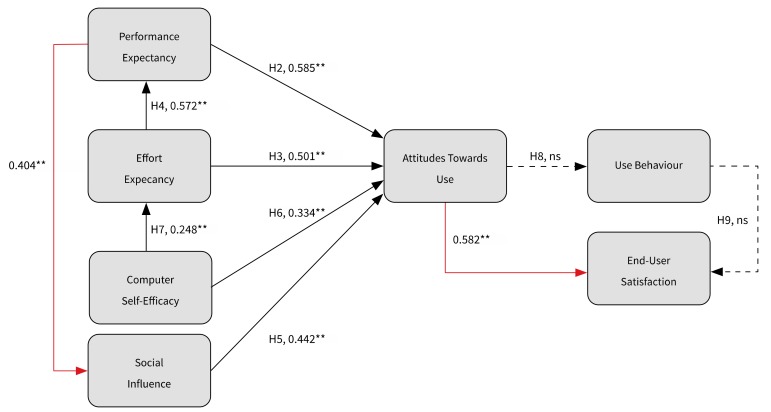
The constructed research model, based on the UTAUT, including newly discovered relationships (red line). ** *p* < 0.001. ns = not significant.

In accordance with the theory, performance expectancy (0.585), effort expectancy (0.501), social influence (0.442), and computer self-efficacy (0.334) correlated positively with the care professionals’ attitudes toward the technology, supporting H2, H3, H5, and H6. Effort expectancy and performance expectancy correlated positively (0.572); the easier the technology was to use, the quicker the care professionals perceived that the technology added value to their work. This finding corroborates findings from Aggelidis and Chatzoglou [8] and Maillet et al. [1] and supports H4. Computer self-efficacy correlated positively with effort expectancy (0.248), which corroborates findings from Maillet et al. [1] and supports H7. No correlation was found between attitudes toward use and use, a finding that does not coincide with relationships suggested by the TRA, TAM, and UTAUT [24]. Thus, H8 was not supported. Unlike findings from Maillet et al. [1], a positive relationship between use and satisfaction was not found, and thus H9 was not supported. After testing the research models and associated hypotheses, a number of additional relationships were investigated. Two new relationships were found. Performance expectancy correlated positively with social influence (0.404), and attitudes towards use and satisfaction also correlated positively (0.582), see Figure 2.

### 4.3. Results of Component Study 3: The Experiences of Care Professionals (End Users)

The care professionals described three positive aspects of working with the devices:

- information can be consulted in a client’s room;
- the devices are easy to use;
- the cameras can be viewed from a distance.

End users also reported some issues for improvement:

- client information that can be consulted while in a client’s room is insufficient; important information is missing, such as wishes concerning resuscitation, contacts, their physician’s contact details, etc.;
- the devices’ operating speed is too low, and it is often impossible to log in. Solutions in these respects are certainly required;
- add report functionality to the devices. The care professionals can only consult information. It would be an improvement if they could use the devices to add information, such as measurements (e.g., blood pressure and weight) and care-specific reports;
- make the devices portable. The devices are mounted in the living room and are not at an ergonomically acceptable height.

Regarding facilitating circumstances, the care professionals were given brief user instructions. Updates concerning the devices were sent by e-mail to both organizations but were not read. The care professionals would like to have structural meetings with technology and policy staff members so that they could have input concerning new functionalities and improvements from a practical viewpoint. The devices changed their way of working, which saved time, but it would save them even more time if the issues for improvement were converted into action. Camera monitoring allowed the care professionals to gauge a client’s situation from a distance, making it easier to prioritize and leading to a better quality of life. One care professional commented, “Thanks to the camera, we can let the clients sleep and do not have to do any nightly physical check-ups anymore.” If information could be reported through the device, fewer mistakes would be made. Measurements are currently written on loose papers, which get lost. If they were able to enter such values directly at a client’s bedside, mistakes could be excluded. The care professionals greatly appreciated the functionality of open calls, partly because it gave an overview of all calls to which they still needed to respond. Regarding retrieval of care protocols, opinions were divided. Some care professionals believed that they should simply know them, and there was thus no need to consult them in a client’s room. Others would appreciate having reference materials available whenever needed.

## 5. Discussion and Conclusions

Using log files, a model based on the UTAUT, and interviews, this study investigates care professionals’ use of advanced technology devices, factors that influence use of the devices, and care professionals’ experiences with them. Component Study 1 suggests that 73% of care professionals use the devices, and 27% do not. Over the entire measurement period, that 27% of care professionals did not use them might be explained by low scores for performance expectancy (i.e., perceived usefulness). Component Study 2 suggests that, on average, this construct is scored lowest (4.72) in comparison to other independent variables (i.e., social influence 4.79; facilitating conditions 4.91; computer self-efficacy 4.74; and effort expectancy 5.48). Performance expectancy is the degree to which users believe that a technology helps them achieve better results. The care professionals perceived that the devices provided insufficient support during care delivery. Interviews conducted during Component Study 3 suggest that this is due partially to the absence of relevant care information, such as resuscitation wishes, contact details, and measurements (e.g., weight, blood pressure, and tension). The care professionals also desired functionality for entry of reports and measures, found the devices too slow, and struggled with access. These factors fall under performance expectancy, as Venkatesh et al. [26] define it. Similar factors are discussed in extant studies as predictors of failure regarding technology use [31,49,50]. The importance of performance expectancy was evidenced in Component Study 2, in which it influenced attitudes toward the devices, explaining 34% of their variance. This finding corroborates results from Taylor and Todd [51], who argue that users attach the greatest value to performance of the technology they use. Kim et al. [10] and Aggelidis and Chatzoglou [8] also demonstrate the importance of performance expectancy during technology use. Care professionals’ attitudes toward technology was influenced by the degree to which they experienced its usefulness (i.e., performance).

Effort expectancy also influenced care professionals’ attitudes toward the devices; their attitudes were influenced by the degree to which they perceived ease of use. Whether the devices were easy to use was reported during the interviews and supported by the care professionals’ perceptions that brief training had provided sufficient information. It was also corroborated quantitatively by high effort expectancy (5.48). Based on the quadrant developed by Keil et al. [52], the care professionals characterized the devices as toys because they were accepted for a brief period but their existence in the long-term is questionable.

This study demonstrates the effect of social influence on care professionals’ attitudes. Since individuals base attitudes on the information and stories of others, this finding was expected; Dwivedi et al. [29] and Nripendra et al. [33] found the same relationship. The interviews revealed that colleagues no longer encouraged each other to use the technology and that the devices were surrounded by negativity, due partially to the fact that the technology did not meet the care professionals’ wishes and needs, and so was perceived as not as useful during delivery of care as other resources were. This implies a new relationship between performance expectancy and social influence.

Component Study 1 suggests that the technology was used an average of six times each day per user. It was mostly used at night and in the mornings, with local client cameras, electronic patient records, and call-and-response logging the most frequently used. Local client cameras were primarily used at night, which was restricted by policy, and the care professionals used them to make virtual rounds. Such rounds allowed the clients to sleep through the night, which added value not only to the clients, but the care professionals. Use of the cameras helped them gauge (emergency) situations and prioritize interventions. When functionality was perceived as adding value and meeting needs, use increased, an argument supported by Hsien-Cheng [49] and Kim et al. [10].

Electronic patient records were largely used during the morning, which is explained by the fact that most care is provided at this time and that client records included a work plan that set the care to be provided and reports on special details. At Organization A, however, electronic patient records were consulted more frequently than at Organization B. The interviews suggested that this is due to care professionals in the extramural context of Organization A needing to register invoiceable time in their clients’ electronic records. This additional obligation increased use, and figures for Organization A were thus misrepresented. Assuming professionals were not obliged to register their time, use of the electronic records would have been similar across the two organizations, and so use of this functionality did not meet expectations. The care professionals reported that electronic client records contributed to work efficiency and quality of care (i.e., fewer mistakes). Such added value is demonstrated by Kim et al. [10] in a study of factors that improve use of electronic patient records among care professionals in hospitals. Since not all desired information in the electronic client records could be used or entered into the device, use of such records was not optimal, especially given workarounds (i.e., paper files) that the interviewees mentioned.

No correlation was found between attitudes toward use and use, or between use and satisfaction. Component Study 1 suggests that use was influenced by the timeframe of duties. The work context (i.e., intramural versus extramural) also affected use, as the interviews revealed. Timeframes and contexts should thus be added to the model as moderators of attitudes and use. Since use of the technology was obligatory at both organizations, it might be an inappropriate outcome. Technology might be used simply out of such obligation, and whenever technology is assessed [1] and its use obligatory [53], it is better to use satisfaction as an outcome.

### 5.1. Limitations and Recommendations for Further Research

In this study, several limitations and opportunities for additional research were identified. A limitation of Component Study 1 concerns the measurement period. Since log files were available only for the last year, quitters over time were not included in the files. Whether the number of non-users was just as high (27%) during initial stages of implementation, or whether low performance expectancy made them quit using the technology, leading to an increase in non-users over time, should be assessed. The cross-sectional design was a limitation of Component Study 2. Further research should focus on a longitudinal approach. This would have provided more insights into use over time and associated changing perceptions. In addition, further research should also link additional variables like leadership [54] to our constructed research model. Another limitation concerns the ordering of questions on the questionnaire, which started with a question of each respondent’s staff number. Had this question been last rather than first, scores on other questions could have been included and the response percentage might have been higher consequently. Respondents who quit completion of the questionnaire when asked to fill in their staff number might have had negative attitudes toward technology use and so did not use it. Component Study 3′s first limitation concerns the number of interviewees (i.e., 6) because this group might not have been representative of the population. Further research should focus on a larger sample among care professionals providing more insights in this industry, since cure-industry results cannot be generalized to the care industry. A second limitation concerns coding, which was conducted by the researchers and thus might have affected reliability, independent co-coding would have been better [39].

### 5.2. Conclusions

This study provides insights into the way care professionals’ advanced technology use can be improved. Results suggest that technology has the potential of being an added value to care professionals during delivery of care. Twenty-seven percent of care professionals did not use it, but users logged in an average of six times a day, and they appreciated the presence of the technology devices in the clients’ rooms. Satisfaction with device use might have been a better outcome to assess in comparison to frequency. The care professionals’ attitudes toward use determined satisfaction with technology use and correlated positively with performance expectancy, effort expectancy, social influence, and computer self-efficacy. Performance expectancy had the greatest influence on attitudes toward use, yet performance expectancy was perceived lowest. Care organizations should focus on improvements to performance expectancy, which can be increased by improving speed, customizing information supplied about a client to the needs of care professionals, and facilitating data entry functionality.

## Figures and Tables

**Table 1:** Summarized overview of the research methods.

Components of This Study			
	Component 1	Component 2	Component 3
	Observable use	Testing of research model	Care professionals’ experiences
Type of research	Quantitative	Quantitative	Qualitative
Research design	Longitudinal	Cross-sectional	Multiple case study
Methods	Logfiles	Questionnaires	Semi-structured interviews

**Table 2:** Characteristics of device users.

	**Total (*n* = 878)**	**Care org. A (*n* = 444)**	**Care org. B (*n* = 434)**
Avg. age (min–max)	42.5 (17–66)	42 (17–65)	43 (18–66)
Avg. contract hrs (min–max)	24.5 (0–36)	23 (0–36)	26 (0–36)
Female (F) Male (M)	92% (*n* = 845) F 8% (*n* = 70) M	94% (*n* = 417) F 6% (*n* = 27) M	91% (*n* = 428) F 9% (*n* = 43) M
	**Total (*n* = 933)**	**Care org. A (*n* = 444)**	**Care org. B (*n* = 489)**
Care aide; level 1 (%)	1	0	1
Care and welfare assistant; level 2 (%)	16	22	11
Individual health care assistant; level 3 (%)	73	63	82
Nurse; level 4 (%)	6	10	2
Nurse; level 5 (%)	2	4	0
Unknown (%)	2	2	3

**Table 3:** Functionalities used per day.

		Morning T1–T4	Afternoon T1–T4	Evening T1–T4	Night T1–T4	Total T1–T4	Avg. Use Per Day, Per Moment
Organization B (*n* = 489)	Client camera on location	115 (5%)	11 (1%)	292 (13%)	1864 (82%)	2281	4.7
	Client camera, location-wide	0 (0%)	0 (0%)	0 (0%)	0 (0%)	0	0 ^A^
	Peripheral camera	2 (2%)	3 (4%)	8 (10%)	68 (84%)	81	0.17
	Peripheral camera, location-wide	0 (0%)	0 (0%)	0 (0%)	0 (0%)	0	0
	Entry camera	5 (31%)	5 (31%)	4 (25%)	2 (2%)	16	0.03
	Electronic patient records	93 (78%)	7 (6%)	16 (13%)	3 (4%)	119	0.24
	Call-and-response logging	12 (9%)	7 (6%)	26 (20%)	82 (65%)	127	0.26
	Vilans protocols	N/A	N/A	N/A	N/A	N/A	N/A
Organization A (*n* = 444)	Client camera on location	80 (9%)	14 _(_2%)	100 (11%)	719 (79%)	914	2.06
	Client camera, location-wide	0 (0%)	0 (0%)	0 (0%)	0 (0%)	0	0 ^A^
	Peripheral camera	3 (2%)	5 (4%)	14 (11%)	101 (82%)	123	0.28
	Peripheral camera, location-wide	0 (0%)	0 (0%)	0 (0%)	0 (0%)	0	0
	Entry camera	3 (2%)	0 (0%)	9 (26%)	21 (62%)	34	0.08
	Electronic patient records	468 (70%)	45 (7%)	119 (18%)	38 (6%)	669	1.51
	Call-and-response logging	9 (10%)	5 (5%)	17 (18%)	61 (66%)	93	0.21
	Vilans protocols	0 ^A^	N/A	N/A	N/A	N/A	N/A
**Total** (*n* = 933)	Client camera on location	185 (6%)	25 (1%)	392 (12%)	2,583 (81%)	3,185	3.4
	Client camera, location-wide	0 (0%)	0 (0%)	0 (0%)	0 (0%)	0	0 ^A^
	Peripheral camera	5 (2%)	8 (4%)	22 (11%)	169 (83%)	204	0.22
	Peripheral camera, location-wide	0 (0%)	0 (0%)	0 (0%)	0 (0%)	0	0
	Entry camera	8 (16%)	5 (10%)	13 (27%)	23 (47%)	49	0.05
	Electronic patient records	561 (71%)	53 (7%)	135 (17%)	41 (5%)	790	0.85
	Call-and-response logging	21 (10%)	12 (5%)	43 (20%)	143 (65%)	219	0.23
	Vilans protocols	0 ^A^	N/A	N/A	N/A	N/A	N/A

^A^ This function was used minimally. N/A, not available.

**Table 4:** Overview of independent variables’ Cronbach’s alpha coefficients.

Variable	*N*	Items	Cronbach’s Alpha
Facilitating Conditions (FCs)	180	4	0.444
Performance Expectancy (PE)	180	4	0.859
Effort Expectancy (EE)	180	4	0.879
Social Influence (SI)	180	4	0.817
Computer Self-Efficacy (CSE)	180	4	0.594
Attitude Toward Use (ATU)	180	4	0.906
